# Correspondence

**Published:** 1888-04

**Authors:** W. H. Pendry

**Affiliations:** Brooklyn, N. Y.


### CORRESPONDENCE.

*To Editors Journal of Comparative Medicine and Surgery:*

GENTS—There is no question that Pasteur, unfortunately for himself, his theories and supporters, has supplied his opponents with plenty of material to work up a strong theoretical opposition by his frequent changes of base regarding his inoculation for hydrophobia; and when we are confronted with this fact, as a supporter of argumentative evidence, as against some of the results of his life's labor, it becomes no easy task to refute some of the assertions made; and the more reading, the more silent arguing, the more impressive does the difficulty of a defence of such a man become; not that I am going to make such an attempt, for the simple reason that I do not for a moment consider I am able to do justice to the defence of such a genius as Pasteur, and therefore leave such a task to able pens; yet my impulsiveness does lead me to try and make some reply to the cynical paper of Dr. Brill. That Louis Pasteur is a marked man of the nineteenth century, even supposing all his theories and results in regard to rabies do not satisfactorily explain themselves to a sceptical opposition, whose name will live long after those of his enemies have been forgotten, is something quite beyond dispute; and while it is quite true that he has given work and opportunity to critics, no act of his has given them the license to use such language as Dr. Brill does. Good honest criticism creates a healthy stimulation of the work criticised. I question if Dr. Spitzka wished us to believe that rabies is a disease of the mind, pure and simple; as I understand, he simply endeavored to demonstrate that by inoculation with simple substances he could produce *similar* symptoms to those described by Prof. Liautard, as resulting from inoculation of a dog said to be rabid. It is true the symptoms were similar, but in the four cases cited by Dr. Brill, he acknowledges the symptoms *varied*, which, it would seem reasonable to suppose, was due to the more or less mechanical injury each received in the operation, as shown even by Dr. Spitzka himself. If we follow the inoculations made in Pasteur's laboratory, where I had the pleasure of noting many, we do not detect any variation whatever; and not only were the symptoms the same, but the results were the same in the same time. Let us, for the sake of argument, give all the weight that can be claimed by Dr. Spitzka, and what does it result in? Simply proving that the Riverdale dog *may* not, after all, have been affected with rabies. No one will be insane enough to claim that all the so-called cases of hydrophobia are the effects of bites of rabid dogs. Rabies, beyond a doubt, is a far more rare disease than is generally supposed, and to try and prove this, I have offered, through the public press, to take charge, free of expense, of any dog said to be rabid, or to have bitten any one; yet I fully believe that such a disease does exist. Looking the results of Pasteur's labors in a fair light, we are obliged to give him the credit of having removed from the public mind the idea that there was no possible remedy for hydrophobia, a phase of his good work that strongly presented itself to my mind, the morning I saw nearly seventy inoculated in his laboratory, every one of whom seemed to believe that they had been rescued from a certain death. Perhaps this belief was their salvation, apart from any result of the inoculation ; but what of that, is not a rescue to such an impression a blessing, irrespective of how it is accomplished, and does not the rescuer deserve some gratitude ? The Commission appointed by the Belgian Government did not throw the matter over, as stated, but simply decided that the "method is not yet sufficiently established," and because the Commission from England endorsed Pasteur's methods, he endeavors to make small of the Commission and its labors.

Are we to tolerate for one moment the idea that a commission composed of such eminent men as Sir James Paget, F.R.S., Dr. Lander Brunton, F.R.S., Dr. George Fleming, F.R.C.V.S., Sir Joseph Lister, F.R.S., Dr. Quain, F.R.S., Sir Hy. Roscoe, M. P., F.R.S., Prof. Sanderson, F.R.S., and Prof. Horsley, F.R.S., would be lead by the nose by one man, as we are asked to believe? Here we have a cluster of medical stars, augmented by a veterinarian of equal eminence that is seldom surpassed, whose endorsement *must* carry weight. The endorsement was not that of one gentleman—Prof. Horsley—as we asked to believe, the report being concurred in by all at an early date, except Sir Joseph Lister, who, for some reason,—what, I did not hear,—caused the report to be delayed, as one of the Commission personally informed me in August, 1886. Dr. Brill attempts to make another point by stating, "There is great *prima facie* evidence that this was chiefly the work of Mr. Victor Horsley, the Secretary of the Commission, (who was no other than Prof. Victor Horsley, F.R.S., and not "Mr.," as stated, no doubt, for effect,) and the one who performed the experiments by which the Commission came to its conclusion." To show the fallacy of this assertion, I quote the conclusion arrived at from advanced sheets of the report of the Commission, which are endorsed August 10, 1886, which was then concurred in by all, except one, as before referred to.

CONCLUSION.

1. The Committee are unanimously of opinion that M. Pasteur's statement that his method of inoculation does prevent the onset of hydrophobia in persons bitten by rabid dogs, is *fully borne* out by the evidence which we *now submit.*

2. The Committee do not recommend the adoption of any definite action upon the above conclusion until they are in a position to report that experiments upon animals now being conducted on their behalf have yielded the same results as those detailed and demonstrated to them by M. Pasteur. The experimental results obtained *so far have confirmed M. Pasteur's statements*, and in particular have proved that the *material he employs for inoculation is actually the virus of rabies* ; but the Committee reserve for a future report the full details and results of this branch of their work.*

Where is the "*great prima facie* evidence ?"

The Committee that was appointed to go to Paris consisted of "Dr. Burdon Sanderson, Dr. Landon Brunton, Sir Hy. Roscoe and the Secretary. * * * The Committee thus delegated had several long interviews with M. Pasteur." This should settle the assertion that Prof. Horsley was a committee of one who went to Paris. The report at that date further says: "The Committee are of opinion that *five per cent.* may be safely taken to represent the average number of cases of hydrophobia *following the bites of rabid dogs.*" So that, even this eminent Committee give it as their opinion that only five out of every hundred persons bitten by dogs *known* to be *rabid* will develop rabies. I think Dr. Brill's assertion that the report does not sustain Pasteur is about as far-fetched as other arguments used by him in trying to prove his "Pasteur fiasco."

BROOKLYN, N. Y. W. H. PENDRY, D.V.S.

* The italics are mine, showing that this opinion was arrived at, without waiting for the results of the experiments being made.

